# The dependent correlation between soil multifunctionality and bacterial community across different farmland soils

**DOI:** 10.3389/fmicb.2023.1144823

**Published:** 2023-04-12

**Authors:** Jing Liu, Zafran Gul Wazir, Guo-Qin Hou, Gui-Zhen Wang, Fang-Xu Rong, Yu-Zhi Xu, Kai Liu, Ming-Yue Li, Ai-Ju Liu, Hong-Liang Liu

**Affiliations:** ^1^School of Agricultural Engineering and Food Science, Shandong University of Technology, Zibo, China; ^2^School of Resources and Environmental Engineering, Shandong University of Technology, Zibo, China; ^3^School of Life Sciences and Medicine, Shandong University of Technology, Zibo, China

**Keywords:** farmland soils, climatic gradients, bacterial community, soil function, multivariate analysis

## Abstract

**Introduction:**

Microorganisms play a critical role in soil biogeochemical cycles, but it is still debated whether they influence soil biogeochemical processes through community composition and diversity or not. This study aims to investigate variation in bacterial community structure across different soils and its correlation to soil multifunctionality. Soil samples were collected from five typical farmland zones along distinct climatic gradients in China.

**Methods:**

The high-throughput sequencing (Illumina MiSeq) of 16S rRNA genes was employed to analyze bacterial community composition in each soil sample. Multivariate analysis was used to determine the difference in soil properties, microbial community and functioning, and their interactions.

**Results:**

Cluster and discrimination analysis indicated that bacterial community composition was similar in five tested soil samples, but bacterial richness combined with soil enzyme activities and potential nitrification rate (PNR) contributed most to the differentiations of soil samples. Mantel test analysis revealed that bacterial community composition and richness were more significantly shaped by soil nutrient conditions and edaphic variables than bacterial diversity. As for soil multifunctionality, soil microbial community level physiological profiles were little affected by abiotic and biotic factors, while soil enzymes and PNR were also significantly related to bacterial community composition and richness, in addition to soil N and P availability.

**Conclusion:**

Cumulatively, soil enzymes’ activities and PNR were greatly dependent on bacterial community composition and richness not diversity, which in turn were greatly modified by soil N and P availability. Therefore, in the future it should be considered for the role of fertilization in the modification of bacterial community and the consequent control of nutrient cycling in soil.

## 1. Introduction

It is widely proven that the biogeographic distribution of the soil microbial community also strongly depends on geographic distance (Ma et al., 2017; Battle et al., 2018) in addition to the contemporary environmental factors (Shi et al., 2020). It is thought that spatial distance is a factor that defined the spread of microorganisms and their variation in community composition across different geographic zones (Mora-Ruiz et al., 2018). As soil microorganisms act pivotal roles in nutrient cycling, productivity maintenance, and carbon sequestration, along with plant growth in an agricultural ecosystem, it could be assumed that soil functions should vary along the spatial distribution of microbial communities across different soils. It is still debated whether soil functions respond to the variation of microbial communities across different soils (Luo et al., 2018), considering an over-proportional role of soil microbial taxa in biological processes (Chen et al., 2020).

Along geographic distance, soil properties and climatic conditions are so distinct that they were supposed to modify the multifunctionality of soil ecosystem through manipulating soil microbial diversity. However, still little is known about how to distinguish the contribution of environmental drivers and microbial communities on soil multifunctionality. Multivariate methods have been well recognized in soil ecosystem research, as they can interpret results with better-summarized information (Ye and Wright, 2010). Principal component analysis (PCA) has been proven sound to analyze the impacts of agricultural practices on microbial community structure and function (Wang X. et al., 2020). Canonical correlation analysis and discrimination analysis also proved potential in identifying the influence of soil chemical properties on microbial community structure and function (Ye and Wright, 2010; Wang Z. et al., 2020).

There is a dependent relationship between soil microbial communities with the identity and number of measured functions (Deltedesco et al., 2020). In our study, bacterial communities and soil functions, including microbial community level physiological profiles (CLPPs), soil enzyme, and potential nitrification rate (PNR), were characterized in all soil samples collected from five typical farmland zones of China. Multivariate analysis methods were applied to answer the following questions: (1) whether soil samples distinguished microbial parameters in addition to climatic and edaphic factors, and (2) whether soil functions were dependent on bacterial communities’ composition or diversity.

## 2. Materials and methods

### 2.1. Sites description and soil sample collection

**Figure 1:**
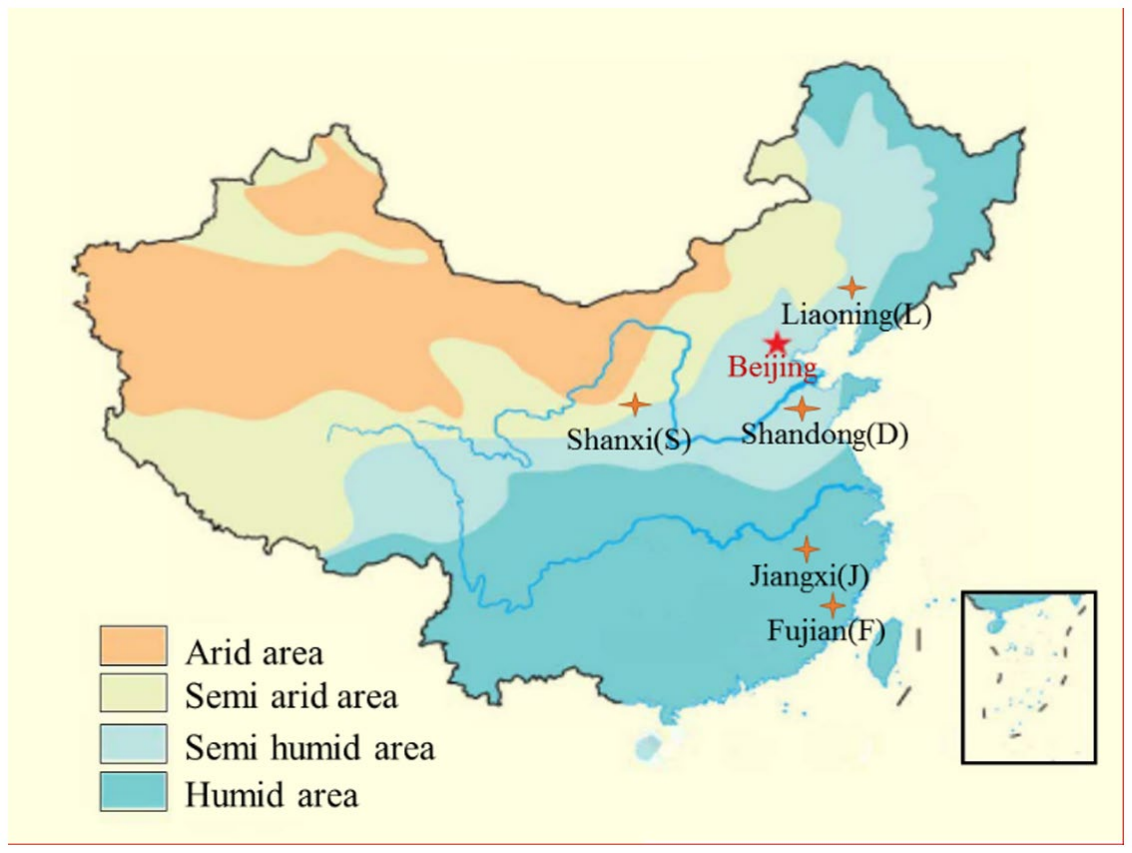
Soil sampling sites’ information.

Five typical farmland soils were collected from Shandong (SD), Shanxi (SX), Liaoning (LN), Jiangxi (JX), and Fujian (FJ), which are located in different farmland zones and climatic zones of China and have experienced long-term intensive agricultural cultivation. Therefore, these collected soils were representatives of soil type, soil management, and climatic conditions (seen in Figure 1 and Table 1). The mean annual temperature and precipitation data for each zone (abbreviated as MAT and MAP, respectively) were offered by China Climatic Data Service Center.^1^ The fertilization data were from the local yearbook of each sampling site.

**Table 1:** Location of sampling, weather conditions, cropping, fertilization, and soil characteristics [listed as mean ± standard error (*n* = 3)].

Parameters	Unit	Sampling sites				
		SD	FJ	JX	LN	SX
Latitude	°N	36.82	25.98	28.35	41.68	34.29
Longitude	°E	118.0	119.38	116.17	123.58	108.07
MAT	°C	14.73	23.33	20.00	10.00	14.67
MAP	mm	925.67	1467.33	1562.67	738.33	662.67
Cropping		Wheat/Peanut	Orchard	Fallow field	Corn	Wheat/Corn
Nitrogen Fertilizer*	kg/hm^2^	251.1	335.2	120.5	192.6	247
Phosphorus Fertilizer*	kg/hm^2^	97.6	180.4	45	64.5	103
Soil type	–	Cinnamon	Red earth	Paddy soil	Meadow soil	loessal soil
pH	–	8.17 (0.04)d	6.04 (0.08)b	4.74 (0.07)a	6.67 (0.07)c	8.55 (0.10)e
Clays	%	5.1(0.08)b	7.64 (1.41)d	4.04 (1.06)a	7.26 (1.52)d	5.94 (0.46)c
OM	g kg^−1^	29.78 (1.46)c	12.91 (0.24)b	7.23 (0.73)a	38.01 (1.75)c	12.67 (1.48)b
TN	g kg^−1^	2.07 (0.19)c	1.27 (0.04)b	1.64 (0.17)c	1.31 (0.05)b	1.15 (0.06)a
TP	g kg^−1^	1.38 (0.04)c	2.25 (0.07)d	1.51 (0.11)c	1.12 (0.03)b	0.70 (0.04)a
LIN	mg kg^−1^	9.53 (0.45)b	11.32 (0.09)c	7.28 (0.29)a	10.81 (0.26)c	15.68 (0.47)d
LON	mg kg^−1^	90.06 (4.37)b	108.71 (3.15)c	73.58 (0.67)a	101.58 (3.40)c	145.15 (11.33)d
LIP	mg kg^−1^	7.55 (0.48)c	4.41 (0.12)b	2.95 (0.09)a	9.46 (0.17)e	8.78 (0.33)d
LOP	mg kg^−1^	11.32 (0.28)c	9.07 (0.10)b	6.84 (0.17)a	14.73 (1.14)d	16.21 (0.23)e

*, is the average value of agricultural fertilization for one crop.

To eliminate the influence of crop growing, surface soil samples (0–20 cm) were collected from a plot without crop growing in November 2019. Three sampling plots with an interval over 50 km were collected for each farmland zone. Five random sites with an interval over 5 km were sampled from each plot and about 2 kg of soil was homogenized as one soil sample after picking out large stones, plant litter, and animal debris. Each sampling site was over 500 m^2^, and triplicate soil samples were collected and mixed as one for each sampling site. The collected soil samples were screened (~2 mm) and then divided into two subsamples: one subsample was stored at ~4°C for microbial analysis in the next 2 weeks, and the other one was air-dried for soil properties analysis.

### 2.2. Soil physicochemical properties’ analyses

Soil pH was measured in a soil–water suspension at a ratio of 1:2.5 mass/volume with a compound electrode on a pH meter (FE22, Mettler Toledo, Shanghai, China). Soil organic matter (OM) was measured by the K_2_Cr_2_O_7_ oxidation method (An et al., 2018). Total nitrogen (TN) and total phosphorus (TP) were determined by colorimetric analysis after being digested with a persulphate solution in an autoclave (Raveh and Avnimelech, 1979; U.S. Environmental Protection Agency, 1993). Soil labile inorganic N (LIN) and P (LIP) were analyzed with a flow injection analyzer (SAN++, Skalar, Netherlands) after extraction with K_2_SO_4_ (Castillo and Wright, 2008) and NaHCO_3_ (Kuo, 1996), respectively. Labile organic N (LON) was the difference between TN and LIN (Castillo and Wright, 2008). Labile organic P (LOP) was the difference between the Kjeldahl P of the NaHCO_3_ extracts and LIP (Castillo and Wright, 2008). Soil chemical properties are shown in Table 1.

### 2.3. Microbial parameters

#### 2.3.1. Soil DNA extraction and bacterial community analysis

The fresh soil samples were transported in iced boxes to Lc-bio Technologie (Hangzhou, China) co., Ltd. for DNA extraction, PCR amplification, sequencing, and taxonomic assignment. Soil DNA was extracted using E.Z.N.A.^®^ Stool DNA Kit (D4015, Omega, Inc., United States). The quality of extracted DNA was determined with a NanoDrop spectrometer (ND 2000, Thermo Scientific, Waltham, MA, United States). The universal primer pair 515F/806R was employed to amplify the V4 region of the bacterial and archaeal 16S rRNA genes. The PCR products were purified and quantified with AMPure XT beads (Beckman Coulter Genomics, Danvers, MA, United States) and Qubit (Qubit4.0, Invitrogen, United States), respectively. The size of the amplicon library was quantified by Illumina 2.0 (Kapa Biosciences, Woburn, MA, United States) with the Library Quantification Kit after being assessed with Agilent 2,100 Bioanalyzer (Agilent, United States). The sequence analysis was conducted on the MisSeq PE250 platform. The operational taxonomic unit (OTU) was defined by Vsearch (V 2.3.4) with a similarity of over 97%, and then each representative sequence was assigned by the Ribosomal Database Project (RDP) classifier. Each microbial library was normalized to the samples with the least sequence number. Alpha diversity and beta diversity were analyzed by QIIME (V 1.8.0) process, and figures were drawn by R (V 3.5.2).

#### 2.3.2. Microbial CLPPs and soil enzymes’ activity

Microbial CLPPs were analyzed according to the procedure of Biolog EcoPlate^™^ (Biolog Inc., Hayward, CA, United States). Briefly, 1 g of fresh soil was extracted with 20 ml of sterilized water after gently shaking for 20 min. After settling for 15 min, 150 μL of supernatant of soil extract was sampled and dispensed into each well of Eco-Plates and subsequently incubated in dark at 25°C for up to 156 h. The absorbance data of each well were recorded regularly at 590 nm using TECAN Infinite^®^ 200 Pro (Tecan Inc., Switzerland). Negative values were considered zero. Average well-color development (AWCD) was calculated according to the references (Garland, 1997; Lewis et al., 2010). The absorbance value of each well was divided by the AWCD and subsequently used to calculate the absorbance value of various C sources as well as for the following principal component analysis (PCA).

Soil urease (URE), phosphatase (PHOS), and dehydrogenase (DHA) were measured by the colorimetric method with a soil enzyme test kit (Cominbio, Jiangsu, China). The added substrate and absorbance wavelength for each soil enzyme are listed in the Supplementary Table S2.

#### 2.3.3. Soil potential nitrification rate

Soil potential nitrification rate (PNR) was measured according to the method described by Liao et al. (2019). Briefly, 20 g soil was spiked with 0.044 M (NH_4_)_2_SO_4_ with a final concentration of 100 mg NH_4_^+^-N/kg soil and then adjusted its humidity to 60% of the WHC followed by a next incubation of 14 days at 25 ± 2°C. Soil (0.100 g) was sampled from each treatment on the 0th and 14th day of incubation, respectively. NO_3_^−^-N in soil samples was extracted with 1 M KCl and determined by the colorimetric method with a SAN++ analyzer (Skalar, Netherlands). The soil PNR computation formula was as follows:


(1) $$PNR=\frac{\omega {\left(NO_{3}{\;}^{-}-N\right)}_{2}-\omega {\left(NO_{3}{\;}^{-}-N\right)}_{1}}{X}$$

where

*PNR* (mg kg^−1^ d^−1^), the potential nitrification rate; *ω*(NO_3_^−^-N)_1_ and *ω*(NO_3_^−^-N)_2_ are the NO_3_^−^-N concentrations (mg kg^−1^) in the soil on the 0th and 14th day, respectively; *X* (day) is the incubation time.

### 2.4. Statistics analysis

A cluster analysis was conducted with Origin Lab 2018 to classify soils by integrated soil environmental factors. A discrimination analysis was conducted to differentiate microbial data across different farmland zones. PCA was used to extract various abiotic and biotic variables into the most important principal components, respectively. A Mantel test analysis was then carried out to investigate the dependent relationship between soil chemical and microbial variables. All data were standardized to zero mean and unit variance before being subjected to multivariate analysis (performed on the Tutools platform).^2^ A one-way analysis of variance (ANOVA) was used to analyze the significant differences among all samples for the selected properties.

## 3. Results

### 3.1. Difference in soil characteristics across five farmland zones

**Figure 2:**
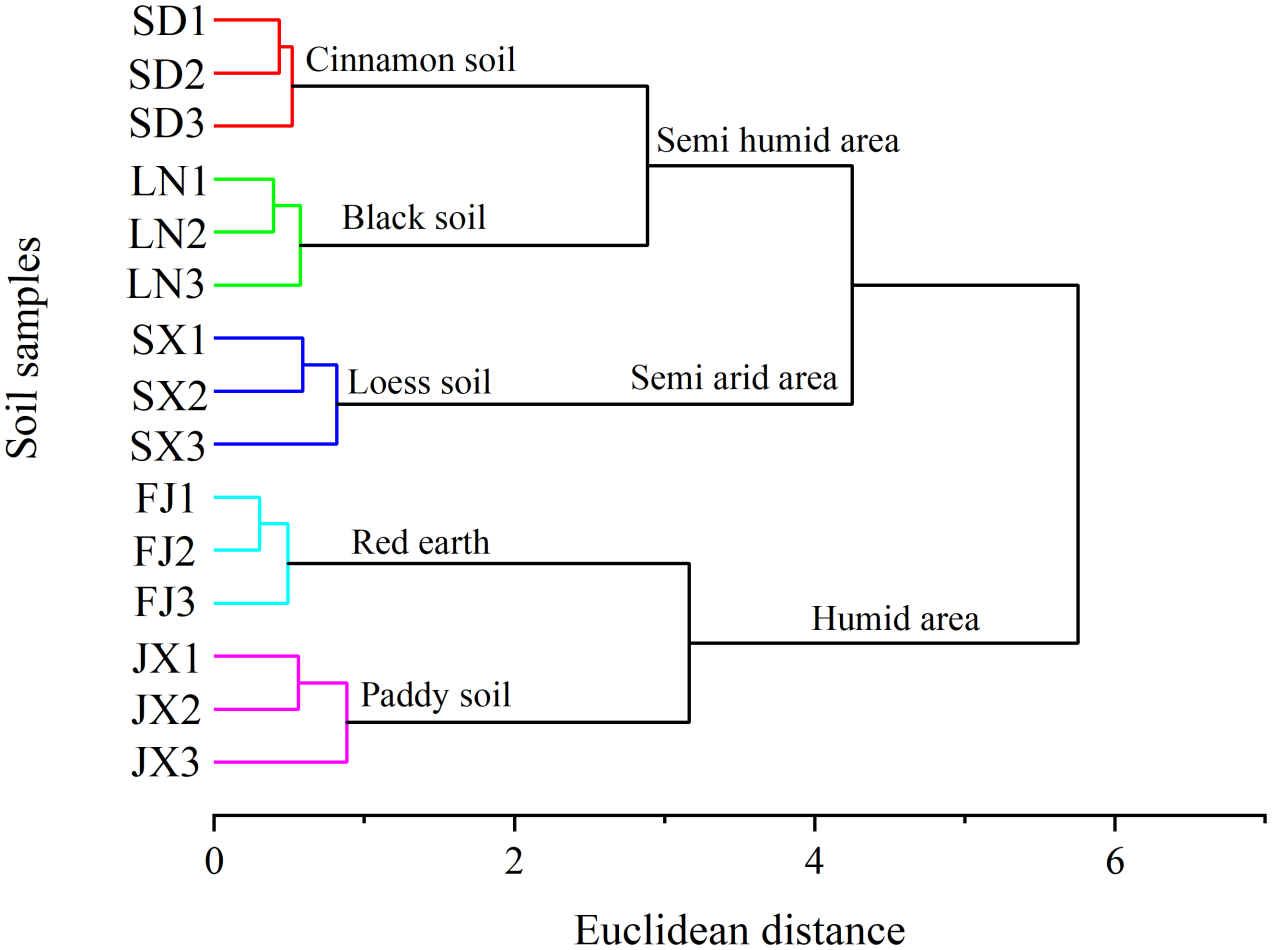
Dendrogram from cluster analysis applied to soil characteristics data (Table 1).

All soil samples revealed a good clustering scheme according to the soil type and climatic gradients (Figure 2, all variables used to construct this dendrogram as shown in Supplementary Table S1). The dendrogram indicated a three-group clustering that cluster 1 contained soils from Fujian and Jiangxi, both located in a southeast humid area of China (Figure 1), cluster 2 only included soils from Shanxi at the central semi-arid area of China (Figure 1), and cluster 3 included soils from Shandong and Liaoning in east semi-humid area, China (Figure 1). Among the investigated soil types, it showed that the cluster of Loess soil was closer to black and cinnamon soil than other two soils belong to red earth and paddy soil, respectively.

**Figure 3:**
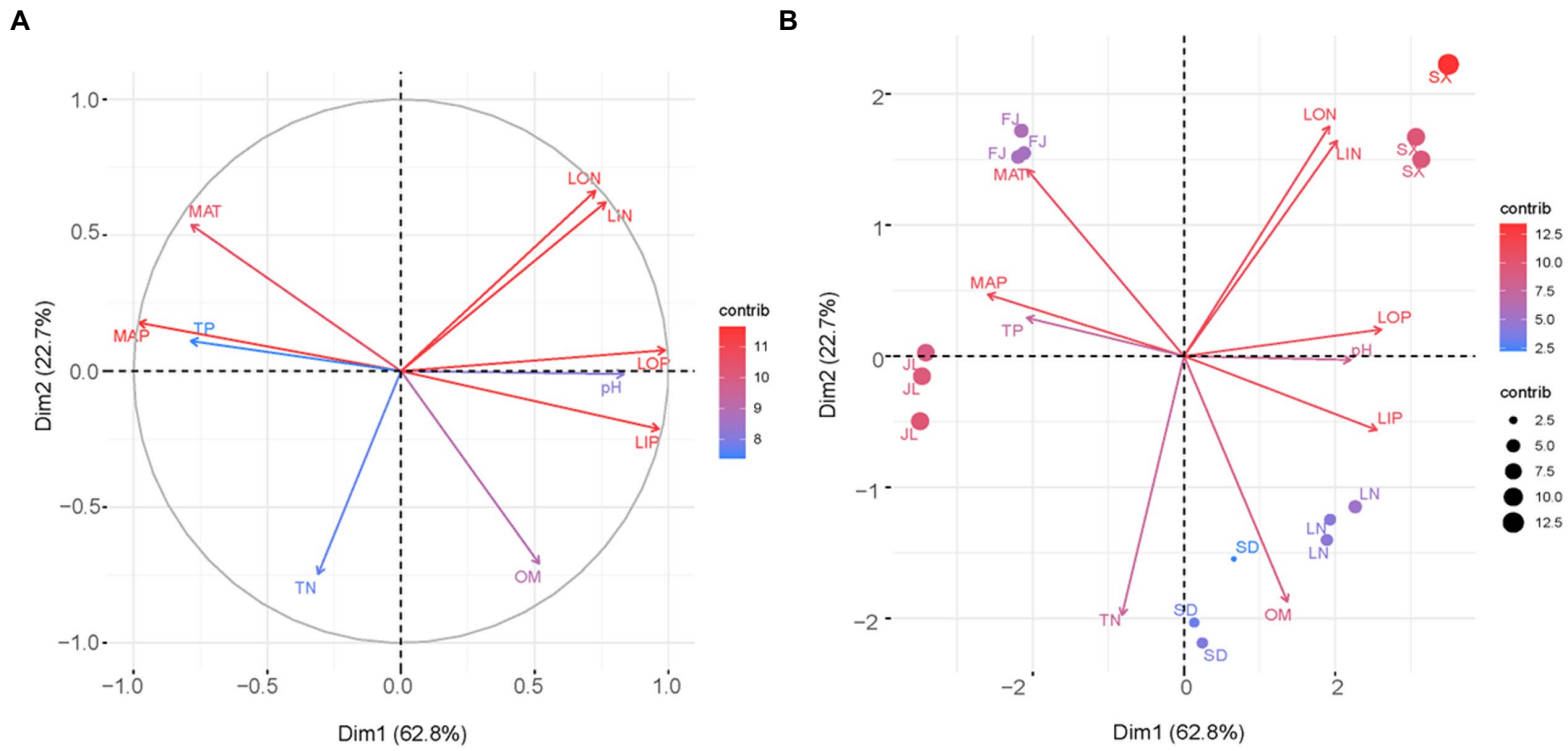
Graphical outputs of principal component analysis using the first two dimensions Dim1 and Dim2. **(A)** Loading plot and **(B)** biplot.

The variables–PCA procedure was used to recognize and extract factors contributing to soil clustering. Although there was a collinear between the variables of LIN and LON as well as MAP and TP, the variables of LIN, LON, LOP, and LIP, combined with MAT and MAP indicated a more important role than TN, TP, and OM in defining the differences among soils (Figure 3A). This was better depicted in the biplot (Figure 3B). That is, the variables LIN and LON were significant in isolating SX soil from the other soil; accordingly, MAT was significant in contributing the separation of FJ soil, respectively, while OM performed a key role in separating SD soil and LN soil.

### 3.2. Difference in soil bacterial community across five farmland zones

**Figure 4:**
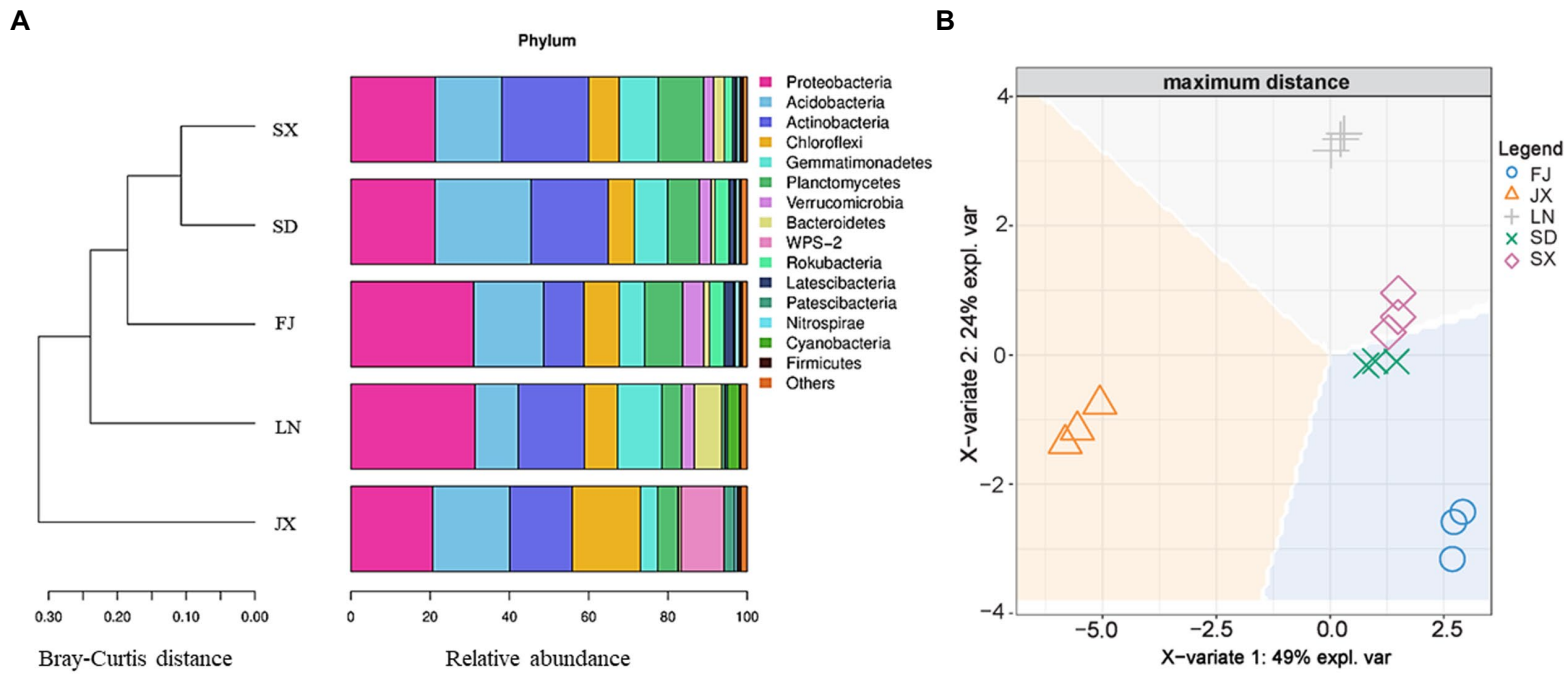
Bray-Curtis distance matrix **(A)** and discrimination analysis **(B)**, where, expl. var is the abbreviation of explanation of variation on bacterial 16S rRNA gene data.

As shown in Figure 4A, all soil samples were similar in bacterial community composition at the phylum level, but they were still divided into three main groups: JX, LN and (FJ, SX, and SD) using the cluster analysis with a Bray–Curtis distance matrix. Similarly, discrimination analysis plots with soil bacterial 16S rRNA gene data also demonstrated that JX soil was significantly separated from the rest soil on the X-variate 1, and LN soil was significantly isolated from FJ, SD, and SX soil on X-variate 2, while SD and SX soil show a good grouping (Figure 4B).

**Figure 5:**
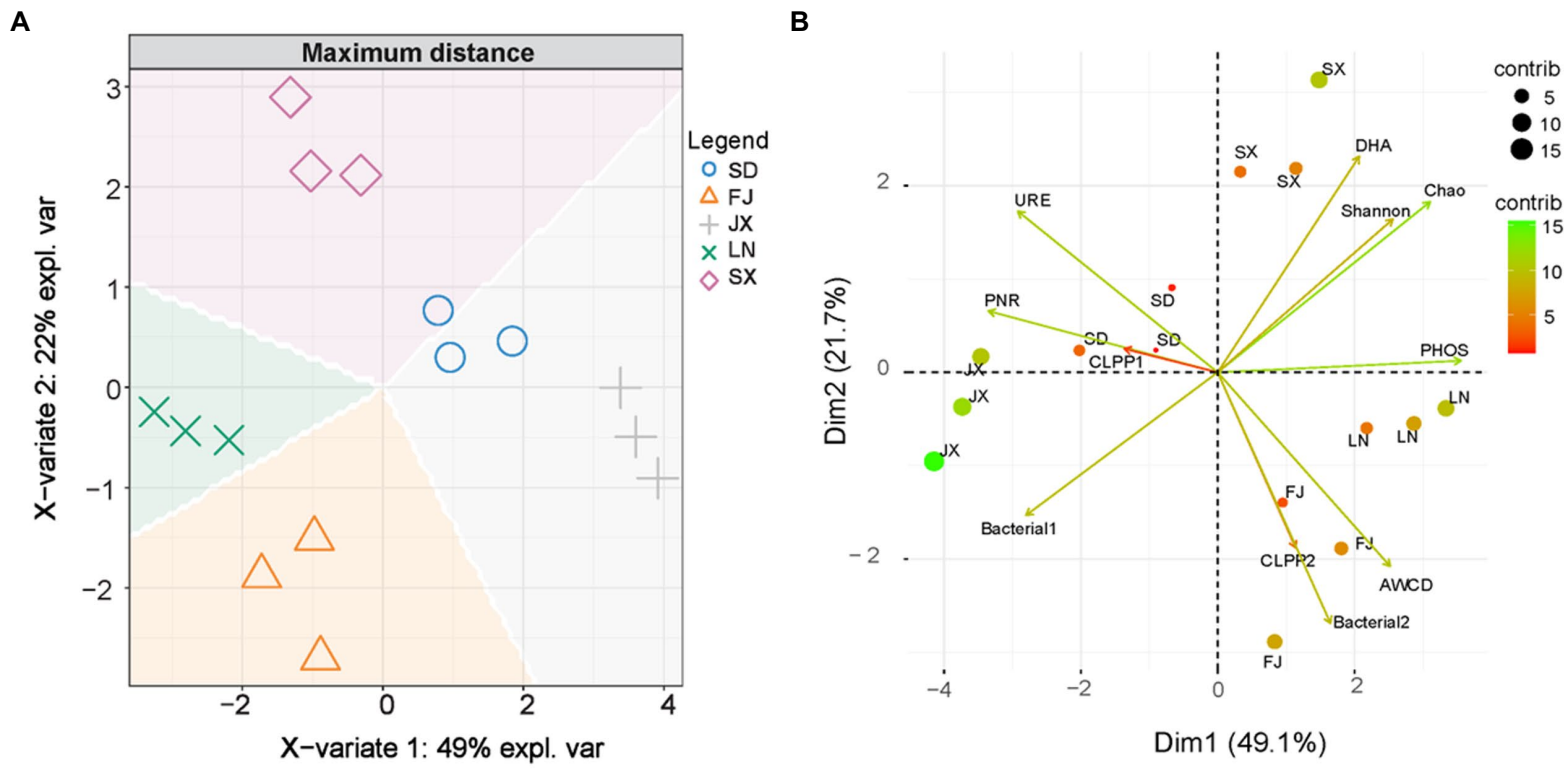
Graphic plots of discrimination analysis **(A)**, where, expl. var is the abbreviation of explanation of variation and principal component analysis **(B)** on soil integrative microbial parameters.

Firstly, principal component analysis was used to create two new variables representing the measured variables of soil microbial community and ecological functions (Table 2). Two variables (Bacterial1 and Bacterial2) were extracted from the original 16S rRNA gene sequences data, which explained 75.52% of the total variance in soil bacterial communities (Supplementary Figure S1). The CLPP1 and CLPP2 extracted from the BIOLOG data set (Supplementary Figure S2) explained 88.27% of the total variance in microbial utilization on various C sources (Supplementary Figure S3). The new variables combined with other microbial parameters (Table 2) were applied for discrimination analysis (Figure 5A), which rearranged relationships between five tested soils. That is, X-variate1 mainly demonstrated the difference between soils from FJ and JX, while X-variate2 exhibited the difference of soil from FJ, LN, and SX, respectively. Biplots (Figure 5B) indicated that Bacterial2, AWCD, and CLPP2 were significant variables isolating FJ soils from rest soils, variables of Chao, Shannon, and DHA contributed to the separation of SX soils from others, and URE and PNR played an important role in the separation of SD soils. However, Bacterial1, representing 50.42% of the variation in bacterial community composition (Figure 4A), appeared no effect on any soil separation.

**Table 2:** Microbial variables of five soils (0–20 cm) listed as mean ± standard error (*n* = 3).

Microbial variables	SD	FJ	JX	LN	SX
DHA (mg kg^−1^ h^−1^)	2.50 ± 0.13b	1.84 ± 0.10a	2.41 ± 0.13b	4.39 ± 0.23c	4.35 ± 0.23c
URE (mg kg^−1^ h^−1^)	45.48 ± 2.37d	11.29 ± 0.59a	41.56 ± 2.16c	10.92 ± 0.57a	30.08 ± 1.57b
PHOS (mg kg^−1^ h^−1^)	64.04 ± 4.72b	80.27 ± 4.18c	22.92 ± 1.19a	89.27 ± 4.65c	77.03 ± 4.01c
PNR(mg g^−1^ d^−1^)	9.78 ± 0.51d	2.70 ± 0.15b	10.70 ± 0.56d	1.84 ± 0.10a	4.31 ± 0.23c
AWCD (OD_590nm_)	1.20 ± 0.06c	1.54 ± 0.08e	0.68 ± 0.04a	1.38 ± 0.07d	0.83 ± 0.04b
CLPP1	0.31 ± 0.47c	0.07 ± 0.03a	0.19 ± 0.14d	0.08 ± 0.11a	0.14 ± 0.07b
CLPP2	0.30 ± 0.07d	0.30 ± 0.20d	0.13 ± 0.04a	0.27 ± 0.12c	0.12 ± 0.11a
Bacterial1	0.12 ± 0.01b	0.29 ± 0.01c	0.48 ± 0.02d	0.02 ± 0.01a	0.09 ± 0.02b
Bacterial2	0.05 ± 0.01a	0.32 ± 0.03c	0.20 ± 0.03b	0.43 ± 0.00d	0.04 ± 0.02a
Chao	1923 ± 100b	2086 ± 108b	1,524 ± 79a	2,317 ± 120c	2,596 ± 135d
Shannon	9.90 ± 0.52a	9.99 ± 0.52a	9.19 ± 0.48a	10.09 ± 0.53b	10.44 ± 0.55b

### 3.3. Dependent relationship between soil environments, bacterial communities, and functions

**Figure 6:**
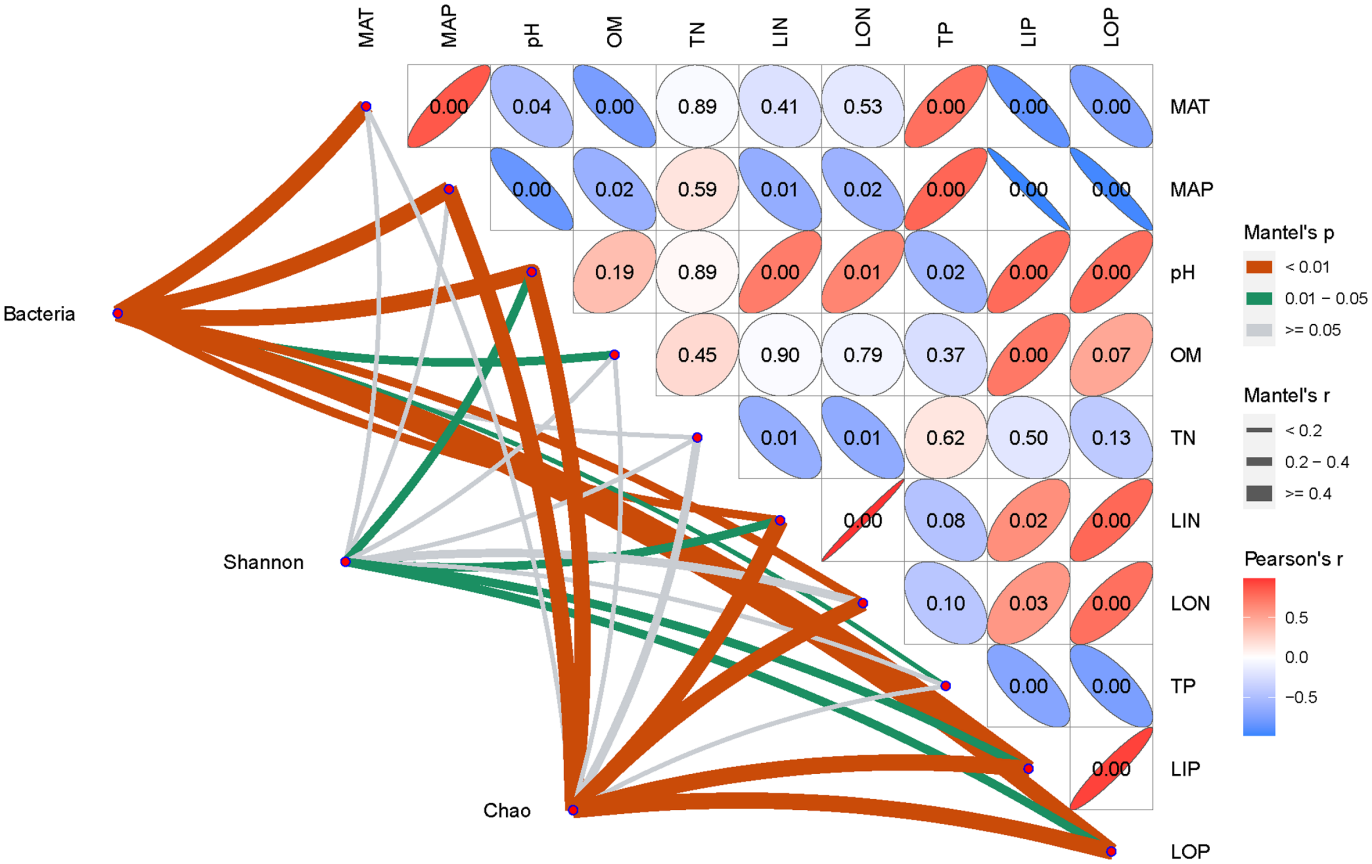
Dependent relationship between soil environmental factors and bacterial communities on Mantel test analysis.

The Mantel test was used to analyze the dependent relationship among soil environmental variables, bacterial community, and ecological functions. As shown in Figure 6, bacterial communities’ composition showed significant relationships with climatic, edaphic factors, and nutrient variables except for soil TN. Similarly, the Chao index, representing bacterial richness, was strongly correlated with most soil environmental parameters at a level of *p* < 0.01, but little related to soil TN and OM. Interestingly, at a level of 0.01 < *p* < 0.05, the Shannon index, which represents bacterial diversity, was only significantly related to soil N and P availability in addition to pH. All of the earlier results indicated that bacterial community composition and richness were shaped by climatic factors and soil nutrient conditions, but bacterial diversity was strongly related to soil N and P availability.

As for soil multifunctionality, the importance of individual soil environmental factors was greatly dependent on the specific soil function, e.g., AWCD, representing integrative microbial activity, had significant relationships with almost all soil chemical properties except TN and TP (*p* > 0.05), while CLLPs, indicating metabolic ability on various C sources, were little affected by soil chemical parameters. Similar to AWCD, soil DHA also had a significant relationship with the majority of soil environmental variables, except pH, TN, and OM. In contrast, soil pH (*r* = 0.30, *p* = 0.015), TN (*r* = 0.52 *p* = 0.006), and LIP (*r* = 0.22, *p* = 0.028) were the only variables that significantly influenced soil PHOS. As important controllers of the soil N process, soil URE only showed a significant relationship with soil TN (*r* = 0.28, *p* = 0.014), while PNRs were strongly dependent on soil TN (*r* = 0.46, *p* = 0.001), LIN (*r* = 0.30, *p* = 0.007), and LON (*r* = 0.28, *p* = 0.008) in addition to soil OM. The earlier results suggest that soil nutrient factors are more important than edaphic/climatic factors (pH, MAP, and MAT) in shaping soil multifunctionality.

**Figure 7:**
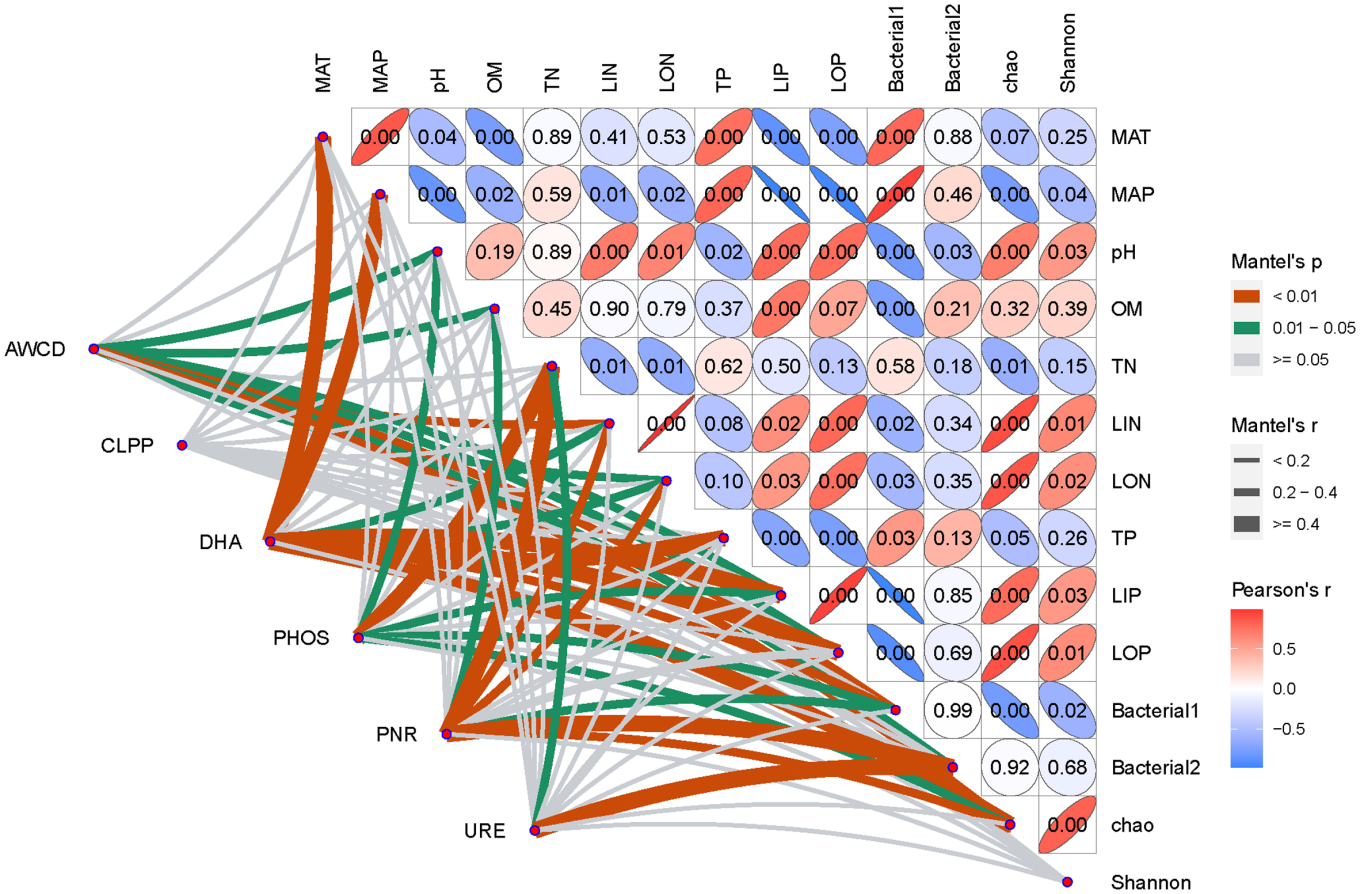
Dependence of soil multifunctionality on soil environmental factors and bacterial communities on Mantel test analysis.

Different from the earlier abiotic soil factors, each tested soil function except for CLPPs was all significantly correlated with bacterial community composition and richness, but not their diversity (Figure 7). In detail, AWCD was all significantly dependent on integrative bacterial community (Bacterial1: *r* = 0.38, *p* = 0.005 and Bacterial2: *r* = 0.23, *p* = 0.032) and richness (Chao, *r* = 0.0.34, *p* = 0.001), while DHA was more strongly dependent on the Bacterial1 community (*r* = 0.36, *p* = 0.006) and richness (*r* = 0.43, *p* = 0.002), which was similar to soil PHOS. In contrast to soil PNR, which is also significantly modified by bacterial community and richness, URE was only greatly dependent on the Bacterial2 community (*r* = 0.72, *p* = 0.001).

## 4. Discussion

### 4.1. Factors shaping soil bacterial distribution across different climatic zones

The soil environment is widely recognized as an important factor in modifying microbial community composition and diversity across distinct ecological zones due to its wide range in soil pH, moisture, temperature, organic matter, and nutrient level (Garcia-Pichel et al., 2013; Fu et al., 2015). In this study, the heterogeneity of the tested soils was depicted by clustering analysis on climatic and chemical variables (Figure 2). Moreover, the Bray–Curtis distance and discrimination analysis on the bacterial 16S rRNA gene also recognized the actual difference in the bacterial community among these soils (Figure 4). In addition, multivariate analysis revealed that soil nutrients availability plaid important roles in distinguished these investigated soils (Figure 3) as well as the inhabited bacterial community (Figures 5, 6), suggesting that soil nutrients levels were also determinative to bacterial community structure and diversity of each tested soil (Xia et al., 2020), in addition to climatic and edaphic factors (Xia et al., 2016; Ding and Eldridge, 2021). Similar results were also reported for other ecosystems and recognized that nutrients availability shaped microbial community structure and function (Ye and Wright, 2010; Griffiths et al., 2011; Geisseler and Scow, 2014; Battle et al., 2018; Shi et al., 2020). This suggests that farming management intensity may be more meaningful to microbial distribution patterns across distinct farmland soils, although soil pH also plays an important role in regulating soil bacterial community and diversity (Figure 6) as previous investigations (Zhalnina et al., 2015; Vázquez et al., 2020; Kang et al., 2021). In other words, bacterial communities are more influenced by regional environmental conditions caused by resulting from intensive farming practices than edaphic factors (Gałązka and Furtak, 2019; Xiang et al., 2020).

### 4.2. Dependent relationship between soil bacterial community and functions

Beyond the recognized influence of soil environment on bacterial communities, increasing investigations are conducted on the effects of edaphic and soil environment on soil functions, e.g., soil enzymes, substrates use profiles and efficiency, and organic matter decomposition. In this study, in contrast to the significant variation across tested soil samples (Supplementary Figure S2), CLPPs were only explained by soil organic matter contents. However, AWCD, representing the microbial integrative function, was significantly linked to most soil chemical properties (Figure 7). This difference may prove that incorporating multivariate ecological functions into an integrative one was more important than only studying the individual relationship between soil parameters and soil function in investigating the effects of edaphic factors on soil functions (Zheng et al., 2019). Moreover, the Biolog Eco-Plates are used to create a metabolic profile of the whole microbial community occurring in different environments (Garland, 1997; Gałązka and Furtak, 2019). In the present study, there was unexpectable to find that CLPPs were not related to bacterial community composition as well as diversity, while a significant correspondence of AWCD was also found to bacterial community and richness (Figure 7). These unexpectable results might be that Biolog Eco-Plates could well depict the habitat characteristics of the original (field relevant) bacterial community (Manzoni et al., 2012), but CLPP from Eco-Plates are lack of immediate links to soil bacterial community in mechanism (Winding and Hendriksen, 2007; Rutgers et al., 2016).

Soil enzymes, originating from various organisms, especially bacteria and fungi, are involved in soil biogeochemical processes under the effects of edaphic factors. Thus, their activities are closely related to soil physicochemical and biological properties (Srinivasrao et al., 2017), in addition to the influences of fertility management, cropping systems, and climatic conditions. Here, along with the significant variation across tested soil samples (Table 2), soil DHA showed a significantly directive relationship with soil bacterial community composition and richness as well as soil nutrients levels, but was little affected by edaphic factors (pH and OM, Figure 7). It is not consistent to the wide recognitions that soil pH and organic contents are of significant factors controlling soil enzymes’ activity (Kang et al., 2009; Agnieszka and Zofia, 2012; Hendriksen et al., 2016; Srinivasrao et al., 2017). Here, a possible reason might be related to soil sampling season, as soil DHA is the most important indicator of soil integrative microbial activity (Agnieszka and Zofia, 2012), and appear to be influenced by temperature *in situ* (Kang et al., 2009). Compared to DHA, soil PHOS was more susceptible to most soil abiotic factors (except TN and TP), but weaker related to bacterial variables (Figure 7). The reason might be that PHOS is mainly from extracellular and less affiliated with microbial biomass compared to DHA (Kang et al., 2009). While in contrast to DHA and PHOS, soil URE only had a significant relationship with Bacterial2 in addition to TN, which might be attributed to the fact that URE only originated from several aerobic bacteria in addition to plants, algae, and fungi (Mekonnen et al., 2021). All of the earlier statements suggest that combined with soil nutrient levels, bacterial community composition and richness play an important role in the control of soil enzymes (de Menezes et al., 2017; Bonner et al., 2018; Zheng et al., 2019). Nevertheless, here no significant associations were found between bacterial diversity and soil enzymes activities (Figure 7), which is not in line with the previous findings (Delgado-Baquerizo et al., 2017a,b; Zheng et al., 2019).

In comparison to three soil enzymes, PNR showed a positive relationship to TN and negative associations with LIN and LON at a significant level of *p* < 0.05. A reasonable explanation is that PNR only participates in the oxidation process of ammonia in soil under the control of the soil nitrifier community (Beeckman et al., 2018). This was also laterally proved by the strong associations between Bacterial2/richness and soil PNR (Figure 7). Nemours studies reported that in addition to OM, soil pH is a critical controlling factor of soil nitrification potential, with a discriminating pH value of 5.3 (Ste-Marie and Paré, 1999), but a weak relationship occurred between PNR and pH in the present study (Figure 7). It suggested that at the ecosystem scale, soil nitrification was not controlled by a single factor but might be an interaction between soil abiotic factors and nitrifier community mostly defined by farming management and soil type (Yan et al., 2018; Zhang et al., 2019; Yang et al., 2021).

## 5. Conclusion

Soil bacterial community and functions were significantly distinct along investigated climatic gradients. The multivariate analysis demonstrated that in addition to edaphic factors, soil nutrients especially their available levels were key factors influencing regional bacterial community composition and diversity as well as soil functions though they were slightly different in strength. Moreover, bacterial community composition and richness were important directive drivers of soil enzymes and PNR, but lack of immediate links to microbial metabolism on various C sources. As a data limitation, the observational correlative results in the present study may be potentially non-causative. Thus, more *in situ* investigation should be conducted on the interactions of environment-microbial community structure–function.

## Data availability statement

The data that support the findings of this study are available from the corresponding author upon reasonable request. All the DNA sequence data in this manuscript are deposited in the GenBank databases, accession number KFXR00000000.

## Author contributions

JL: data curation and original draft preparation. ZG: English improvement and modification. G-QH and F-XR: data curation and experiment investigation. Y-ZX, KL, and M-YL: visualization and editing. A-JL: conceptualization and methodology. H-LL: reviewing and validation. All authors contributed to the article and approved the submitted version.

## Funding

This study was supported by the Shandong Province Natural Science Foundation under grant nos. ZR2020ZD19 and ZR2022YQ34, and the Natural Science Foundation of China (NSFC) under grant nos. 42077129, 41877122, and 42177403.

## Conflict of interest

The authors declare that the research was conducted in the absence of any commercial or financial relationships that could be construed as a potential conflict of interest.

## Publisher’s note

All claims expressed in this article are solely those of the authors and do not necessarily represent those of their affiliated organizations, or those of the publisher, the editors and the reviewers. Any product that may be evaluated in this article, or claim that may be made by its manufacturer, is not guaranteed or endorsed by the publisher.

## Footnotes

^1^ http://data.cma.cn

^2^ http://www.cloudtut.com
