# Supplementary material for: The dependent correlation between soil multifunctionality and bacterial community across different farmland soils
Source: Front Microbiol. 2023 Apr 12;14:1144823. doi: 10.3389/fmicb.2023.1144823 (PMC10132505; doi:10.3389/fmicb.2023.1144823)
Supplement: Supplementary file 1 [file Data_Sheet_1.pdf]

Supplementary information for

The dependent correlation between soil multifunctionality and bacterial community across different farmland soils

Jing Liu[1], Zafran Gul Wazir[1], Guo-Qin Hou[1], Gui-Zhen Wang[1], Fang-xu Rong[1], Yu-zhi Xu[2], Kai Liu[2], Ming-yue Li[2], Ai-ju Liu[2]✉, Hong-liang Liu[3]✉

[1] School of Agricultural Engineering and Food science, Shandong University of Technology, Zibo, 255049，P R China

[2] School of Resources and Environmental Engineering, Shandong University of Technology, Zibo, 255049，P R China

[3]School of Life Sciences and Medicine, Shandong University of Technology, Zibo, 255049，P R China

Table S1 The location of sampling and soil characteristics

Parameters	Unit	Sampling sites				
		SD	FJ	JX	LN	SX
Latitude	° N	36.82	25.98	28.35	41.68	34.29
Longitude	° E	118.0	119.38	116.17	123.58	108.07
MAT	℃	14.73	23.33	20.00	10.00	14.67
MAP	mm	925.67	1467.33	1562.67	738.33	662.67
Cropping		Wheat/Peanut	Orchard	Fallow field	Corn	Wheat/Corn
Soil type	--	Cinnamon	Red earth	Paddy soil	Meadow soil	loessal soil
pH	--	8.17 (0.04)	6.04 (0.08)	4.74 (0.07)	6.67 (0.07)	8.55 (0.10)
Clays	%	5.1(0.08)	7.64 (1.41)	4.04 (1.06)	7.26 (1.52)	5.94 (0.46)
OM	g kg^{-1}	29.78 (1.46)	12.91 (0.24)	7.23 (0.73)	38.01 (1.75)	12.67 (1.48)
TN	g kg^{-1}	2.07 (0.19)	1.27 (0.04)	1.64 (0.17)	1.31 (0.05)	1.15 (0.06)
TP	g kg^{-1}	1.38 (0.04)	2.25 (0.07)	1.51 (0.11)	1.12 (0.03)	0.70 (0.04)
LIN	mg kg^{-1}	9.53 (0.45)	11.32 (0.09)	7.28 (0.29)	10.81 (0.26)	15.68 (0.47)
LON	mg kg^{-1}	90.06 (4.37)	108.71 (3.15)	73.58 (0.67)	101.58 (3.40)	145.15 (11.33)
LIP	mg kg^{-1}	7.55 (0.48)	4.41 (0.12)	2.95 (0.09)	9.46 (0.17)	8.78 (0.33)
LOP	mg kg^{-1}	11.32 (0.28)	9.07 (0.10)	6.84 (0.17)	14.73 (1.14)	16.21 (0.23)

Table S2 The substrates, absorbance wavelength of the supernatant and the results expressed of the

Urease and Dehydrogenase enzyme assays.

Enzyme activities	Substrates	Absorbance wavelength	Results ($\mu g \cdot d^{-1} \cdot g^{-1}$)
Urease	Urea	578	NH_3-N
Phosphatase	SPP	660	C_6H_5OH
Dehydrogenase	TTC	485	TPE

SPP: Sodium Phenyl Phosphatase

Fig.S1

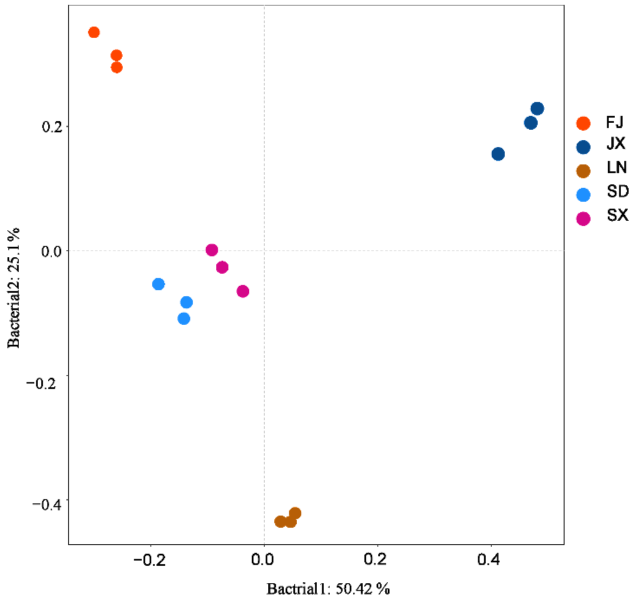

Fig.S1 Score plots of principal components analysis on original 16S rRNA gene sequences data

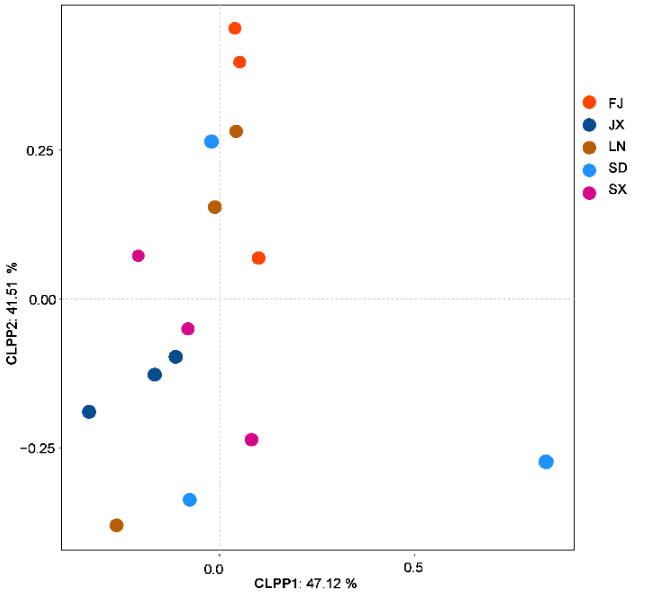

Fig.S2 Score plots of principal components analysis on original Biolog data

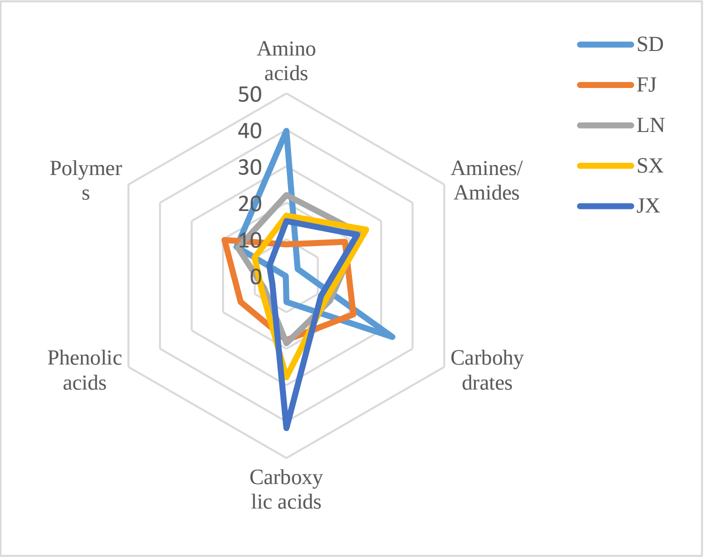

Fig.S3 Microbial metabolic characteristic on various carbon sources on Biolog Eco-plate^{TM} in different soils
